# Home Range and Ranging Behaviour of Bornean Elephant (*Elephas maximus borneensis*) Females

**DOI:** 10.1371/journal.pone.0031400

**Published:** 2012-02-08

**Authors:** Raymond Alfred, Abd Hamid Ahmad, Junaidi Payne, Christy Williams, Laurentius Nayan Ambu, Phua Mui How, Benoit Goossens

**Affiliations:** 1 Borneo Species Programme, World Wildlife Fund-Malaysia, Kota Kinabalu, Sabah, Malaysia; 2 Borneo Conservation Trust, Sabah Wildlife Department, Kota Kinabalu, Sabah, Malaysia; 3 Institute for Tropical Biology and Conservation, Universiti Malaysia Sabah, Kota Kinabalu, Sabah, Malaysia; 4 Borneo Rhino Alliance (BORA), Institute for Tropical Biology and Conservation, Universiti Malaysia Sabah, Kota Kinabalu, Sabah, Malaysia; 5 World Wildlife Fund Asian Rhinoceros and Elephant Action Strategy, World Wildlife Fund-International, Gland, Switzerland; 6 Sabah Wildlife Department, Kota Kinabalu, Sabah, Malaysia; 7 School of International Forestry, Universiti Malaysia Sabah, Kota Kinabalu, Sabah, Malaysia; 8 Danau Girang Field Centre, Sabah Wildlife Department, Kota Kinabalu, Sabah, Malaysia; 9 Organisms and Environment Division, School of Biosciences, Cardiff University, Cardiff, United ingdom; Australian Wildlife Conservancy, Australia

## Abstract

**Background:**

Home range is defined as the extent and location of the area covered annually by a wild animal in its natural habitat. Studies of African and Indian elephants in landscapes of largely open habitats have indicated that the sizes of the home range are determined not only by the food supplies and seasonal changes, but also by numerous other factors including availability of water sources, habitat loss and the existence of man-made barriers. The home range size for the Bornean elephant had never been investigated before.

**Methodology/Principal Findings:**

The first satellite tracking program to investigate the movement of wild Bornean elephants in Sabah was initiated in 2005. Five adult female elephants were immobilized and neck collars were fitted with tracking devices. The sizes of their home range and movement patterns were determined using location data gathered from a satellite tracking system and analyzed by using the Minimum Convex Polygon and Harmonic Mean methods. Home range size was estimated to be 250 to 400 km^2^ in a non-fragmented forest and 600 km^2^ in a fragmented forest. The ranging behavior was influenced by the size of the natural forest habitat and the availability of permanent water sources. The movement pattern was influenced by human disturbance and the need to move from one feeding site to another.

**Conclusions/Significance:**

Home range and movement rate were influenced by the degree of habitat fragmentation. Once habitat was cleared or converted, the availability of food plants and water sources were reduced, forcing the elephants to travel to adjacent forest areas. Therefore movement rate in fragmented forest was higher than in the non-fragmented forest. Finally, in fragmented habitat human and elephant conflict occurrences were likely to be higher, due to increased movement bringing elephants into contact more often with humans.

## Introduction

The Asian elephant inhabits many types of habitat throughout its range, including closed canopy tropical rainforest in Peninsular Malaysia, Sumatra and Borneo. These three land masses were part of Sundaland, an extension of the continental shelf of South-East Asia, most of which now lies under the South China Sea [1]. In recent decades, commercial logging of the forests was followed by large-scale conversion of logged forests to agriculture and by the establishment of settlements in these three parts of Sundaland. As a result, the available elephant habitat was reduced, forcing a large percentage of these animals to migrate to other areas with a change in their feeding behavior since the natural habitat was affected and the food supply was depleted [2].

Home range is defined as the extent and location of the area covered annually by a wild animal in its natural habitat [3]. Studies of African and Indian elephants in landscapes of largely open habitats have indicated that the size of their home range is determined by a combination of factors including ongoing human disturbance [4], food supply, seasonal changes, availability of water sources [5] and the existence of man made barriers such as canals and also habitat loss [6]. For the Asian elephant, there is considerable evidence that indicates home ranges are influenced by the availability of suitable habitat [2]. Sukumar [2] also emphasized that the more diverse a habitat is for an Asian elephant, the smaller the required home range as elephants would be able to meet their varied needs within a relatively small area. To our knowledge, the home range size for the Bornean elephant has not been reported, probably due to the difficulty of tracking individuals in the forest. The only available information on the Asian elephants' home range in Sundaland rainforests was reported by Olivier [7] to be 59 to 167 km^2^ in Peninsular Malaysia.

As the home ranges of introduced feral animals may be significantly different to animals that had evolved in that habitat, it is important to emphasise that the origin of the Bornean elephant is still controversial, despite the publication of a molecular study indicating the genetic distinctiveness of the Bornean elephant and its derivation from Sundaic stock [8]. The authors also claimed independent evolution of the Bornean elephant for some 300,000 years since a postulated Pleistocene colonization and recognized it as native to Borneo and as a separate evolutionary significant unit. However, it seems unlikely that a taxon assumed to be present in Borneo for more than 300,000 years, and therefore subject to evolutionary pressures, presents only one maternal lineage as compared with orang-utans or proboscis monkeys which harbour several maternal lineages and are also present on the island since the Pleistocene [9]–[10]. More strikingly, there have been no authenticated or confirmed finds of Asian elephant in any controlled excavation, including the Niah cave (Sarawak) or the Madai cave (Sabah, within the species' present range) although other large ungulates (*Rhinocerus sondaicus* and *Dicerorhinus sumatrensis*, *Tapirus indicus*) were excavated. Cranbrook *et al.* [11] took into account such facts and postulated a different theory: elephants from Java were sent to Sulu at the end of the 14^th^ century as a gift between Sultans, proliferated on the island and subsequently provided the founder members of the existing population of northeast Borneo. However, it remains unclear when and how many founders were translocated to Borneo by the Sultan of Sulu. Elephants were reported to be present on Sulu until the beginning of the 19^th^ century but they were finally exterminated by 1850 [11]. Therefore, Borneo may have been the refuge of the Javan elephant and *Elephas maximus borneensis* the descendant of *E. m. sondaicus*. This being the case, these elephants have only inhabited Sabah for between 500 to 600 years.

The successful application of satellite tracking systems to follow large wildlife species in order to determine the size of their home range and their movement patterns in different habitats has been possible since the early 1980's [12]–[13]. Particularly for elephants, it has been used on African savannah elephants (*Loxodonta africana africana*) in several countries such as Namibia [14]–[16], Botswana [17], Mozambique [18], Cameroon [19], Zimbabwe [20], Kenya [21]–[22] and Tanzania [23], and on African forest elephants (*L. a. cyclotis*) in Central African Republic and Congo [24]–[25]. Most of these studies looked at home range size, habitat use and preferences, migration and activity patterns, and seasonal movements. For example, de Beer & van Aarde [16], using satellite GPS collars, discovered that landscape heterogeneity and water distribution determined elephant home range location and size, leading to management measures for the elephants in conservation areas. In Asia, satellite tracking devices have only been used in India to determine movement patterns and habitat use of one individual male elephant [26] and in Peninsular Malaysia to track the movements of two translocated individuals, a female and a male [27]. However, scientists have also used VHF telemetry to study the ranging patterns of 10 elephants in southern Sri Lanka [28]. Table 1 shows the estimated home ranges of the Asian elephant in several locations in Asia. Due to some variation in the methodology (especially to gather the location of the elephants in the forest) and the degree of completeness of the gathered data, the estimated home ranges for the Asian elephant were about 100 km^2^ to 300 km^2^, this should be regarded as a minimum home range in a non-fragmented forest landscape.

**Table 1:** Estimated home ranging for Asian elephant based on previous studies in Asia and using Minimum Convex Polygon (MCP).

Source	Size of home range	Method	Tracking Method	Remarks
Khan [33]	166.6 km^2^	MCP	Visual Observation and radio tracking	A group of adults in Peninsular Malaysia
Khan [33]	84.3 km^2^	MCP	Visual Observation and radio tracking	Sub-unit that comprises young elephants in Peninsular Malaysia
Khan [34]	313 km^2^	MCP	Visual Observation and Foot tracking	In Peninsular Malaysia
Olivier [7]	167 km^2^	MCP	Radio tracking	In primary forest in Peninsular Malaysia
Olivier [7]	59 km^2^	MCP	Radio tracking	In secondary forest Peninsular Malaysia
Sukumar [2]	105 km^2^ to 115 km^2^	MCP	Radio and GPS tracking	In India
Sukumar [2]	170 km^2^ to 320 km^2^	MCP	Radio and GPS tracking	In India

This paper presents data on the free ranging Asian elephant of Borneo in their natural habitat using satellite GPS telemetry (satellite GPS collars). It also presents data on their movement patterns obtained from GPS data locations in conjunction with field observations of the known individuals. In addition, we estimated (i) the home ranges for the Bornean elephant in non-fragmented and fragmented forests, and (ii) the minimum period of tracking needed to determine the home range. We finally ascertained the typical patterns of elephant movement over time and determined how fragmented forest and habitat conditions affect the movement and how it may contribute to human-elephant conflict.

## Results

### Capturing and Tracking Elephants

Five female elephants (two large sub-adults and three adults) were captured and collared between 10 June and 17 July 2005.

**Figure 1:**
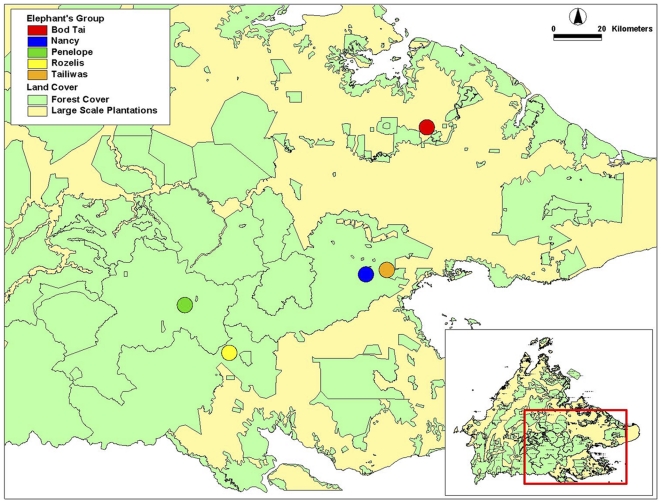
Location of the successful capturing and collaring of the elephants, Sabah Malaysia.

Table 2 identifies the location and details of capture of each elephant and the name given to the herds. Locations of the captures are shown in Figure 1. Only two of the elephant herds (Nancy and Bod Tai) were tracked for over a full year in Ulu Segama-Malua Forest Reserves and Lower Kinabatangan Wildlife Sanctuary. Table 3 provides the details on period and tracking performances using satellite GPS collars for each collared elephant. The performance of the satellite GPS collars used to track the elephants for one year ranged from 33.8–46.2% reliability. The elephants' home range derived from this study takes into consideration the performance of the satellite GPS collars. The home range calculated in this study should be considered as a minimum home range, since the availability and predictability of food resources have always been strongly implicated as having the most important effect on ranging.

**Table 2:** Summary of elephant collaring and locations.

No	Location name	Existing forest type(s)	Successful collarings (date)	Name given to elephant's herd	Age/Sex
1	Batu Timbang	DF/HF		-	-
2	Kalabakan	DF	13 June 2005	Rozelis	Large immature female
3	Taliwas	DF	22 June 2005	Tailiwas	Adult female
4	Ulu Segama Malua	DF/UF	23 June 2005	Nancy	Adult female
5	Lower Kina-batangan	DF/FSF/MF	7 July 2005	Bod Tai	Large immature
6	Gunung Rara	DF	17 July 2005	Penelope	Adult female
7	Deramakot	DF	-	-	-

DF for Dipterocarp Forest, HF for Heath Forest, UF for Ultramafic Forest, FSF for Freshwater Swamp Forest, MF for Mangrove Forest.

**Table 3:** Summary of tracking periods and tracking performances using satellite GPS collar for each elephant herd.

Name of herd	Estima-ted herd size	Date of collaring	Tracking record dates	Overall duration of tracking	Number of GPS location obtained	No of days with GPS location obtained	% of tracking (Daily tracking)
Rozelis	12	13 June 2005	13 June–4 July 2005	21 days	16	12 days	57.1%
Tailiwas	45	22 June 2005	22 June 2005–14 January 2006	216 days	58	49 days	22.7%
Nancy	23	23 June 2005	23 June 2005–21 June 2006	364 days	165	123 days	33.8%
Bod Tai	65	7 July 2005	7 July 2005–7 July 2006	366 days	277	169 days	46.2%
Penelope	43	17 July 2005	17 July–4 November 2005	110 days	32	29 days	26.4%

### Sizes of Home Range

#### a) Home Range

**Figure 2:**
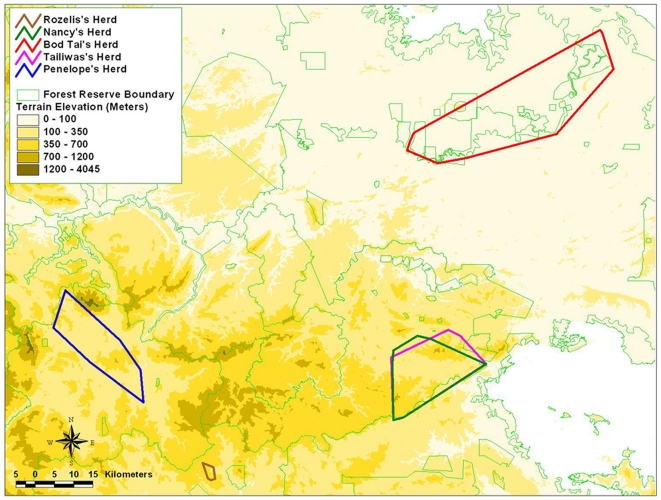
Home range pattern generated by the elephant herds based on MCP method.

**Figure 3:**
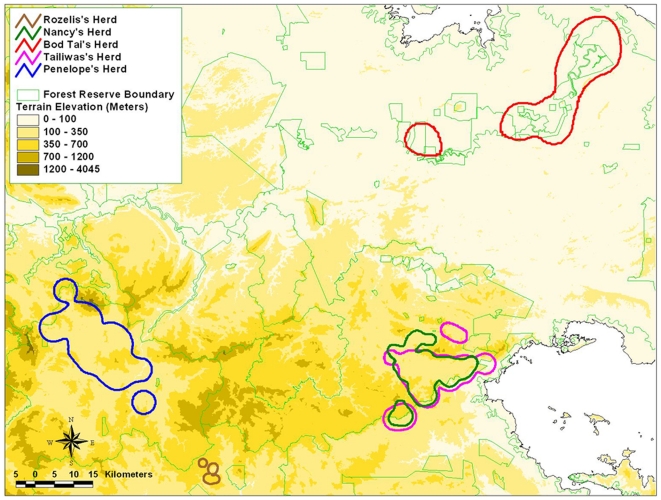
Home range pattern generated by the elephant herds, based on HM methods.

The home range patterns computed using MCP and HM methods are shown on topographical maps (Figures 2 and 3), while the numerical results are summarized in Table 4.

**Table 4:** Home range of each elephant based on Minimum Convex Polygon (MCP) and Harmonic Mean (HM) home range estimation methods, and average monthly ranging generated using MCP and HM.

Elephant Herds	Rozelis	Tailiwas	Nancy	Bod Tai	Penelope
Tracking Period (days)	21	216	364	366	110
Home Range (km^2^) using MCP	11.90	316.20	291.54	778.62	248.88
Core Area 1 (km^2^) using HM with 65% isopleths	7.06	118.00	80.49	112.11	113.77
Core Area 2 (km^2^) using HM with 75% isopleths	10.44	155.48	113.38	141.44	171.78
Core Area 3 (km^2^) using HM with 85% isopleths	15.55	205.07	152.99	256.46	252.67
Average Monthly Ranging (MCP)		50.25	50.12	120.86	59.48
SD for Monthly Ranging (MCP)		31.17	21.63	147.56	14.74
SE for Monthly Ranging (MCP)		13.93	6.52	42.60	8.51
Average Monthly Ranging (HM)		103.77	110.27	277.56	191.25
SD for Monthly Ranging (HM)		27.25	35.49	426.27	118.33
SE for Monthly Ranging (HM)		13.63	10.70	123.05	68.32

As Rozelis was only tracked for a short period of time, we did not use the data in the analysis. Tailiwas and Nancy herds shared the same forest area and had a home range of 330.41 km^2^ and 244.97 km^2^ respectively (HM method, with 95% isopleths). Although Tailiwas was only monitored for 216 days, the home range of her herd was bigger than Nancy's herd. The home range for Penelope's herd covered about 414.8 km^2^ over a period of 3.5 months.

The home range for Bod Tai's herd calculated by the MCP was 778.62 km^2^. This figure is misleading because it incorporates extensive areas of oil palm plantations and permanent swamp forest, where elephants may not be able to access because the plantations are secured or protected by electrical fences, and they are known to rarely if ever enter swamp forest. In comparison, the HM method (95% isopleths) estimated the home range to be 593.02 km^2^. This is considered to be a much more accurate and reliable home range area. Although Bod Tai's herd lives in marginal and fragmented habitat (isolated from the main elephant habitat of central Sabah, elephants have to travel periodically through plantations and villages, in a generally swampy and flood prone region), parts of the habitat provide fast-growing grasses and other nutritious plants on fertile soils of the Lower Kinabatangan that are favoured by the elephants.

The amount of suitable habitat for elephants in Lower Kinabatangan may now be too small and too fragmented to support an elephant population in the long term. Although the elephant population in this area appears to be increasing, based on the frequency of infant elephants seen in all herds, it will not be able to expand, as they are isolated by roads, plantations and human settlements.

#### b) Monthly Ranging

The monthly ranging of the monitored elephants was analyzed based on the location data gathered from the satellite GPS collar for each month. The monthly ranging that is generated using MCP and HM methods is presented in Table 4.

The monthly ranging of the elephants in the non-fragmented forest, based on three different individuals was between 50 to 60 km^2^ (using MCP method) and 103 to 192 km^2^ (using HM method, 95% isopleths). The monthly ranging of the elephants in the fragmented forest, based on one individual was 120.86±42.60 (using MCP method) and 227.56±123.05 (using HM method, 95% isopleths). It would seem that the monthly ranging of the elephant in the non-fragmented forest is smaller compared to the one in the fragmented forest.

**Figure 4:**
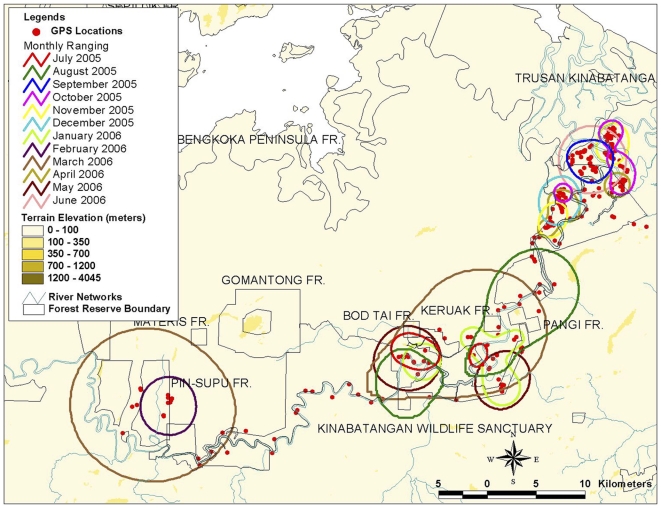
Monthly ranging for BodTai's herd in fragmented forest (using Harmonic Mean 95% isopleths).

**Figure 5:**
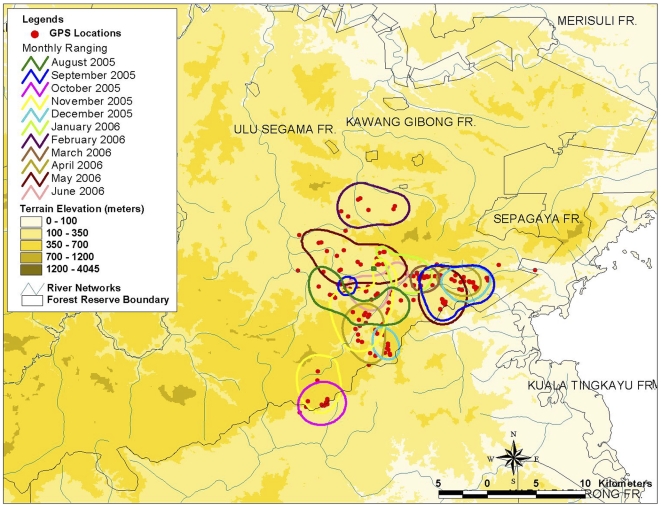
Monthly ranging for Nancy's herd in fragmented forest (using Harmonic Mean 95% isopleths).

The highest monthly ranging for the elephant in the fragmented forest was recorded during March 2006, when Bod Tai's herd moved along a narrow riparian strip in Lower Kinabatangan, and utilized patches of fragmented forest. Figures 4 and 5 show the monthly ranging for Bod Tai's herd and Nancy's herd for one year (generated using the Harmonic Mean with 95% isopleths).

Using the Harmonic Mean with 95% isopleths, the monthly ranging for Nancy's herd covered 110.27±10.70, which was similar to Tailiwas's herd range of 103.77±27.25. However, the monthly ranging for Penelope's herd was twice as big, estimated at 191.25±68.32.

### Elephant Movement Patterns

The satellite tracking data provided an opportunity to analyze the rates at which the five collared individuals moved within their home ranges in different habitat types. The speed of the elephant movements was computed by generating the average distance taken by the monitored elephant in one full day, based on the available GPS location data and the time between successive locations (Table 5).

**Table 5:** Mean daily rate of elephant herds' movements.

Name of elephant herd	Number of days with consecutive GPS locations	Distance (Km)	Mean rate of movement (Km/day)	Standard deviation (SD)	Standard error (SE)	% CV
Rozelis	5	5.49	1.10	1.06	0.47	43%
Tailiwas	19	23.81	1.25	1.04	0.24	19%
Nancy	22	27.93	1.27	0.95	0.20	16%
Bod Tai	111	200.49	1.81	1.92	0.18	10%
Penelope	8	12.07	1.51	0.82	0.29	19%

Rozelis's movement rate (based on distance per day) during the first day was observed to be high at 2.86 km – presumably due to the stress of being captured and collared. Field observations of Rozelis showed that she was isolated from her herd for the first two days after collaring. From the third day her movement rate varied from 0.75 to 1.20 km per day. She joined her herd after one week.

Tailiwas's movement rate on the first day was about 1.56 km. The longest distance moved (4.29 km) was detected on 15th September 2005, when the herd moved into Devata village and was chased away by villagers. During the 12-month study period the herd entered the village at least twice. The distance moved per day for this herd ranged from 0.75 to 2.90 km.

Nancy's movement rate during the first day after collaring was about 0.52 km. In general, the movement rate for this elephant herd was similar to Tailiwas' herd, except that Nancy's herd entered the village area only once. The distance moved per day for this herd ranged from 0.30 to 3.80 km.

Bod Tai's herd moved at least 6.7 km in the first day after collaring. The distance moved per day for this herd ranged from 0.30 to 9.50 km. Bod Tai's herd moved more than 9.52 km one day after the herd was chased away from Sukau village. Movement of this elephant herd was high when they moved along a narrow corridor of natural vegetation along the banks of the Kinabatangan River bordering an oil palm plantation and through swamp forest, where elephant's food is limited and where travel for a large mammal was difficult.

The movement rate of Penelope and her herd was higher when they were crossing from upland forest to lowland forest using an abandoned logging road in Gunung Rara Forest Reserve, traveling at least 2.90 km per day. The distance moved per day for this herd ranged from 0.75 to 2.90 km. This higher movement rate is probably due to the herd needing to access water sources which are only available in a lowland forest. The elephants will use abandoned logging roads as their migratory routes especially in the upland or higher terrain, due to the higher abundance of food sources (i.e. grass), which contain higher water volume.

The monitored elephants exhibited a mixture of movement rates in different types of forest. For example, they moved more frequently and over larger distances whenever they were utilizing fragmented forests. In the fragmented forests of the Lower Kinabatangan, changes in monthly movement patterns were expected as human settlements occur and barriers such as electric fences have been erected, altering movement patterns. Harassment by humans also influenced the direction and speed of movement. In non-fragmented forests elephants were less erratic in their observed movement pattern.

**Figure 6:**
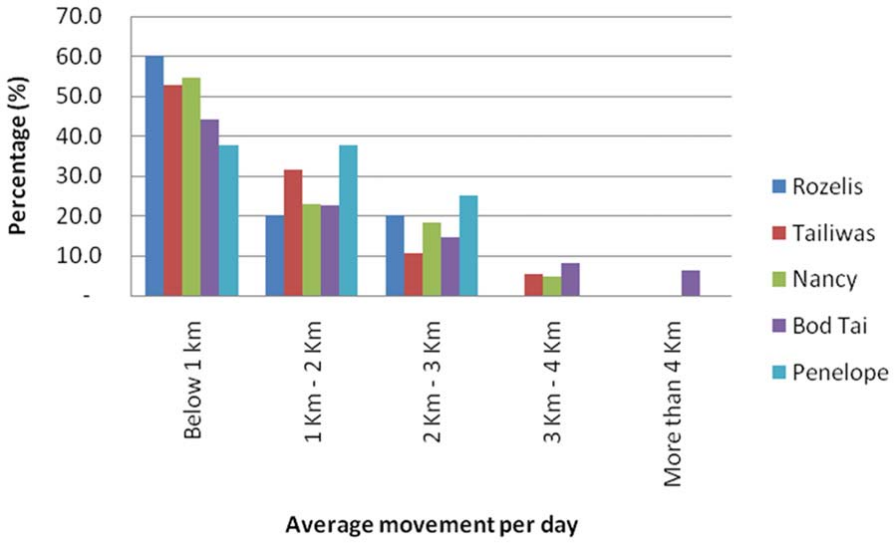
Percentage of the elephant herd's movement per day.

Figure 6 shows the daily movement pattern for the five elephants. In non-fragmented dipterocarp forest the average movement rate for the elephants was 1.30±0.10 km per day and the maximum distance moved in a day was 2 km. In contrast, in highly fragmented forest the rate of travel was 1.81±0.18 km (40% higher) per day and maximum distance moved in a day was 4 km.

### Habitat Utilization

**Figure 7:**
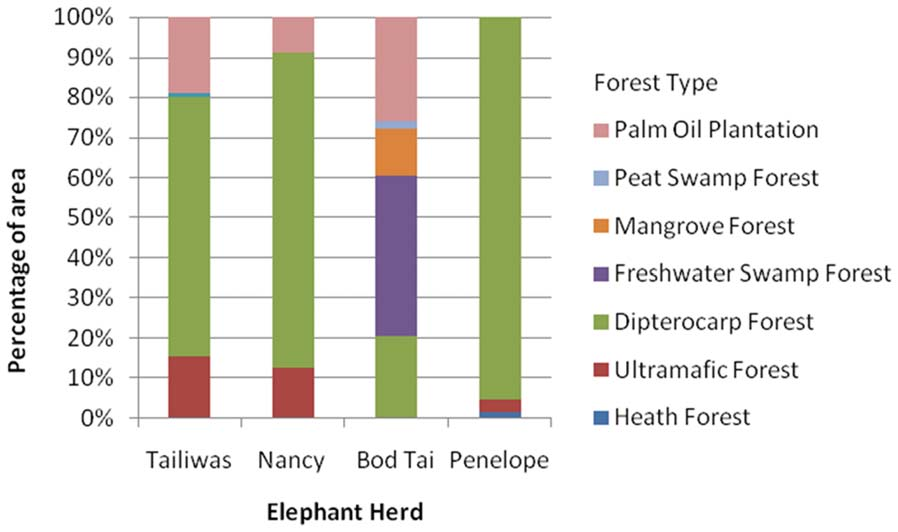
Percentage of areas with different forest types, utilised by the monitored elephants.

**Figure 8:**
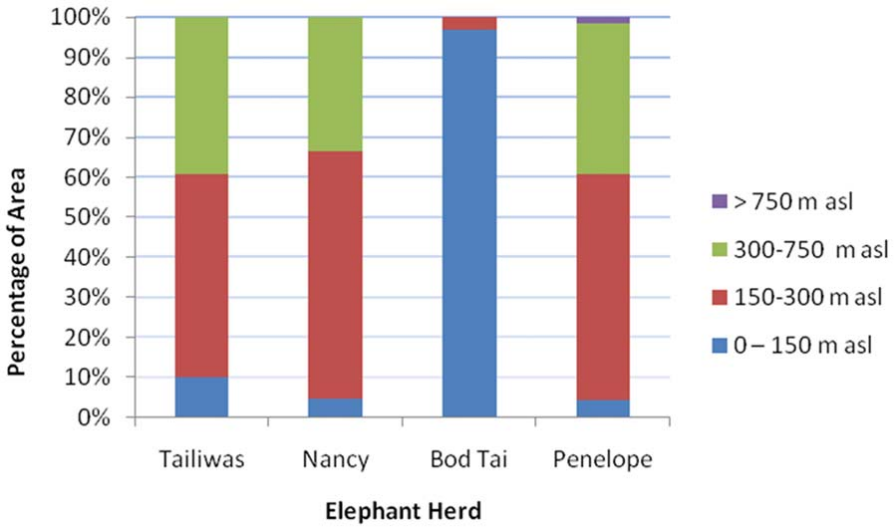
Estimated percentage of areas with different altitude classes, utilised by the monitored elephants.

Figure 7 shows the percentage of the forest habitat type utilised by each herd within its home range (determined by MCP) and Figure 8 shows the percentage of areas with different altitude utilised. The elephant herds spent the majority of their time below 300 m of altitude.

## Discussion

### Estimation of the Home Ranges for Bornean Elephants in Fragmented and Non-fragmented Habitats

Based on the field observations, it is considered unlikely that the elephants spent much or any time in ultrafamic forests and in palm oil plantations. The former presents limited water sources and elephant food plants in addition to the toxic heavy metals present in leaves (derived from the metal rich ultrafamic soils), while in the latter there are no plants suitable for foraging. The elephant herds spent the majority (ranging between 65%–95%) of their time in dipterocarp forest, except for Bod Tai's herd in the Lower Kinabatangan where most of the habitat utilised by the elephants was freshwater swamp forest. The fact that most of the previous lower land dipterocarp forests that were located within the Kinabatangan habitat landscape were already converted into oil palm plantations might explain the results for this region. In rainforest areas, the home range size of family groups was suggested to be larger in primary forest (up to 167 km^2^), where food plants are less abundant, than in secondary forest (up to 59 km^2^) [7]. Contrarily, the current study shows that the herd's home range size in secondary forest was greater (248 km^2^ to 317 km^2^) than the one previously reported [7]. The difference between the current results and those from preceding researchers [7] may be due to several factors [29], particularly by the availability of suitable forest habitat and permanent water. Different forest types and conditions provide different diversity and density of food resources, including the availability of permanent water, all of which influence the home range size. Tailiwas' and Nancy's herds were tracked in the Ulu Segama Forest Reserve which is below 300 meters above sea level (asl) and has been largely logged. Currently the area is composed of degraded dipterocarp forest with sparse tree cover, and of secondary forest growing on soils derived from sedimentary rocks. Penelope's herd was also found to utilize the same forest type and condition; this could be due to habitat disturbance from logging activities being carried out in Gunung Rara Forest Reserve during the study period.

Based on the estimated home range, the data from the current study shows that the home range for the collared elephants in fragmented forest was double that of collared individuals in the non-fragmented forest habitat. This could reflect the elephants' difficulty to find their daily resource requirements in an environment that has been greatly altered. The other factors influencing the home range size are likely to be the conflict between elephants and humans (settlements and agricultural activities) and the construction of barriers (channels and fences). For instance, elephants were recorded to move a longer distance after being chased by humans; it is likely that their usual movements were altered to avoid certain areas or their home range has been altered by human activity. This situation was experienced and recorded by the tracking team on the ground during the field observations of Nancy's and Tailiwas' herds, on a day the herds were chased by the villagers in Sepagaya village (located at the south-east of Ulu Segama-Malua Forest Reserves). The same pattern of movement was also recorded by the tracking team on the ground during the field observations of Bod Tai's herd when they reached Batu Putih village in Lower Kinabatangan region.

### Estimation of the Minimum Period of Tracking Needed to Determine the Home Range

The current results showed that collared elephants living in logged dipterocarp forests completed their annual home range (maximum home range) within a period of less than six months. The home range for Tailiwas' in the first four months (316.20 km^2^) was very similar to Nancy's, after 12 months of movement (291.54 km^2^). No disturbances, such as logging activities, were recorded during the study in Nancy's home range. Based on his study in Peninsular Malaysia, Olivier [7] also suggested that, due to environmental constancy, tracking for six months would provide a minimum home range for a herd of elephants. This would be more accurate than if the elephants were only tracked through a season, although home range size would undoubtedly continue to increase with a longer monitoring period.

### Typical Patterns of Elephant Movement in (i) Non-fragmented Forest; (ii) Fragmented Forest; and (iii) in Different Levels of Disturbance

Elephants were shown to live mostly in lower forest with altitude ranging from 150–300 metres. It is likely that low altitude forests are favoured for a number of reasons not the least of which would be proximity to permanent water sources (elephants require between 100–225 litres of water a day; [2] and [5]), and ease of movement. In addition, the constant climatic conditions throughout the year and throughout the study area are unlikely to have a great seasonal effect on food availability. It also can be assumed that the relative availability of food remains constant in each forest area provided the type and quality of the forest is the same. A higher availability of elephant food in non-disturbed forest should be manifested in a higher standing of food (density and diversity) than in disturbed forest (i.e. logging is in progress). There should also be an increased predictability of food sources, with food either being less patchily distributed or be in higher concentration, or both, than in disturbed forest.

The differences in behavioral ecology due to the difference in forest condition (i.e. fragmented and non-fragmented forest) is particularly clear for the elephant's herds when there are statistically significant differences between mobility indices (movement rate) (see Table 5). It is reasonable to assume that elephant food in the fragmented forest will be depleted more rapidly, to a point where movement is necessary, and that elephant herds would need to move further until feeding could be resumed, especially when compared to non-fragmented forest. Assuming that equal size herds feed at approximately the same rate and respond to the same level of depletion in food resources, then it is also reasonable to assume that herds of comparable size would be more mobile in fragmented forest than in non-fragmented forest. This is supported by the collared elephant data that showed there was greater movement per day by animals in the fragmented forest compared with those in the continuous forest.

This study also indicates that habitat disturbance had the greatest effect on the elephants' movement. In Gunung Rara Forest Reserve, logging activities were carried out during the tracking period and elephants moved greater distances than in forests that were not being logged. It is likely that food plant sources would have been depleted and that the elephants would have been affected by the presence of humans and noise from the logging activities [7].

## Materials and Methods

### Tracking the Elephant Herds

Global Positioning Systems (GPS) mounted inside a tracking collar and combined with a satellite communication transmitter provide invaluable information on elephant location and movement. This technology allows locations to be transmitted to the satellite, and compiled in the computer anytime of the day without the need for difficult and expensive fieldwork.

The satellite GPS collars were supplied by Africa Wildlife Tracking, Inc, Pretoria, South Africa. They consisted of a GPS device, an internal aerial, a transmitter, and a battery. A counterweight on the collar ensured that the functioning parts of the unit stayed on top of the elephant's neck to allow a clear path of signal reception from the satellites. The entire collar weighed between 13–15 kg, a minimal weight to be carried by a three-ton (3,000 kg) elephant [30].

Each tracking device reads its own position via GPS satellites. This information is sent either to one or four communication satellites, or to a cellular network. The information is then transmitted to the globaltrack server (http://www.globaltrack.com) where it is processed and can be downloaded by the user. A GPS can be used to determine the exact geographic location with an accuracy that is much higher than can be achieved with a map and compass. The accuracy of positions determined with a GPS ranged from ±1 m to ±15 m.

The duty cycle of the units was set to download three GPS fixes per 24-hour period, one every eight hours (0600, 1400 and 2200). Each satellite GPS collar contained a conventional “Very High Frequency” (VHF) transmitter that was used to track the elephants on the ground. The radio frequencies were ranging from 140 to 150 Hertz.

During the satellite tracking period, elephants were also tracked on foot. Ground truthing of elephant locations were undertaken in all habitat types. In Kinabatangan, where the transmission of the GPS signal from the collar was about 366 days, approximately 77% of the GPS locations received (169 days with GPS location) were verified on the ground. Other location data could not be verified due to the difficulty of accessing the areas (e.g. swampy area).

Informal interviews with the oil palm workers were conducted to determine if the elephants were entering the plantations. It was revealed that the elephant herds entered the oil palm plantations occasionally (at least once or twice a year), and stayed in the forest adjacent to the oil palm plantation for a 1–2 week period.

### Capturing and Collaring Elephants

All animal work has been conducted according to Malaysian and international guidelines. During a six-week period from June to July 2005, two large immature and three adult female elephants were tracked, captured and collared with the satellite tracking devices in five separate areas in Sabah. Locations of the captures are shown in Figure 1, and the type of habitat in each capture location is described in Tables 6 and 7. The elephant herds were tracked in the early morning (between 0400 to 0630) on foot until the herds of adult females were found. The ranger armed with a tranquilizer gun, darted one of the adult female elephants and then tracked the darted elephant until it was sedated.

**Table 6:** Description of the habitat found in each capture location.

Habitat Description	Name of elephant herd
Industrial timber plantation and heavily logged dipterocarp forest combined with one patch of undisturbed dipterocarp forest.	Rozelis
Regenerating logged dipterocarp forest with patches of primary dipterocarp and ultramafic forest	Tailiwas
Regenerating logged dipterocarp forest with patches of primary dipterocarp and ultramafic forest	Nancy
Degraded dryland and freshwater swamp forests, scrub, riverside forest and mangrove, oil palm plantation, villages	Bod Tai
Logged dipterocarp and heath forest and primary upland dipterocarp forest (Logging activities were active in this area during the tracking period)	Penelope

**Table 7:** Description of the forest types.

Forest Type	Description
Heath Forest (HF)	The tree canopy averages between 5 and 30 meters tall, but is fairly homogeneous in a particular area. There are few big lianas, but many slender ones. Trees of the family Myrtaceae usually predominate.
Ultramafic Forest (UF)	UF varies greatly in structure and species composition, but is usually dominated by species rare or absent from other nearby forests. On hill slopes, UF tends to have a very even rather low canopy in comparison with DF. The only common factor in all UF is that it develops on soils derived from ultramafic rock.
Dipterocarp Forest (DF)	The original DF is tall forest which is characterized by the presence of a fairly high biomass density of large trees of the family Dipterocarpaceae, and often Leguminosae and Lauraceae. The original DF has been very heavily disturbed and nearly all of plants in DF are secondary growth of different species composition from that in the original forest.
Freshwater Swamp Forest (FSF)	Plant species composition in FSF varies greatly, and may be locally diverse, or dominated by one species. The tree canopy is generally rather open, but with extensive patches of low scrub in the most poorly-drained areas.
Mangrove Forest (MF)	Mangrove is characterized by a relatively few species of trees growing in coastal areas inundated with seawater. Nipah is a palm, which forms pure species stands where salt and fresh water mix.
Peat Swamp Forest (PSF)	The structure of PSF varies greatly, ranging from low, stunted vegetation to forest resembling DF. Floral composition is equally variable. The habitat is characterized by a layer of peat (slightly decomposed plant material), 0.5 to over 20 m deep developed on marine alluvium.
Palm Oil Plantation (POP)	POP's are planted on a large scale with species of palm tree, for the purpose of producing oil palm. Most of the DF has been replaced by POP in Sabah especially in the south-eastern part.

Mature female elephants, of a healthy appearance and more than seven feet in height (over about 30 years of age), were selected for two reasons: firstly, the study aimed to focus on herds with young, which are led by a mature female, and secondly, to reduce potential stress that may have been caused by the weight of collars. The sedative used was Iliul Xyllazil-100, it took effect within 30 minutes after the first dart was fired successfully, and the effect lasted for 30–40 minutes. Once sedated, the elephant was kept in a standing position by using small poles pointed at the back of the elephant's ears and the collar with the tracking device was attached around the elephant's neck. To minimize unnecessary time sedated, an antidote (Reverzine) was administered as soon as collaring was completed, normally taking less than a minute to take effect. In some cases, the rest of the herd stayed nearby throughout the collaring, while in other cases, they fled. The collared elephant returned to its herd either immediately or within a few days.

The success of immobilizing and collaring these elephants depended on finding elephant herds with the right characteristics (e.g. sex, age and health requirements) within the time period for fieldwork and the ability of the ground staff to track the herd in the forest. Seven different areas were identified to conduct the collaring of wild elephants, but only in five areas were elephants successfully collared. Of those five areas, four areas were located within the large block of predominantly logged but non-fragmented forest cover in central Sabah, and one area was located in a fragmented floodplain landscape (see Figure 1).

### Data Compilation and Analysis

The data obtained from the satellite tracking was compiled by using the web-based application developed by Global Track (www.globaltrack.com). The GPS collar device was programmed to transmit the GPS locations every 8 hours (0200, 1000, and 1800). During the daytime, elephants were sighted resting in the forest between 0900 to 1500, and during this time very few signals were transmitted due to forest canopy cover. GPS locations were only transmitted when the elephants were in the open areas, such as in riverine (this was especially during the evening) or logged forest. In order to analyze habitat utilisation, only home range data that was collected for a period of more than three months was analysed.

The size and shape of the collared elephant's home range was calculated by using a Geographical Information System (GIS) software that applies two methods, namely Minimum Convex Polygon (MCP) method and Harmonic Mean (HM) method. MCP involves drawing the smallest polygon using GIS that contains all the location points for the tracked elephants. It is considered that MCP is not suitable to be used for calculating elephant home ranges. Osborne [31] highlights that MCP is heavily influenced by outliers and the range increases as more fixes are added. The other limitation of this method is that it does not show the area of most activity. HM method of analysis attempts to define a harmonic mean centre of activity. Range boundary defined using HM method can be represented by the isopleths enclosing the smallest matrix values [32]. Hence, the centre of activities could be highlighted by HM method and concentrations of activity are identified as the core areas if they are at the periphery of the range.

In this study, the HM method was used to determine the size of the home range, including the core area. The core area for the elephant was identified with 65% isopleths, while the centre of activity was identified with 85% isopleths, and home ranging or area of distribution was identified with 95% isopleths.

Forest type and altitude range covered by a herd were determined. Table 6 provides a brief description of the forest in each capture location, and Table 7 lists the forest types in each study area.

### Conclusion

This study provides provisional evidence that elephant herds in Sabah occupy a minimum home range between 250 km^2^ to 400 km^2^ in the non-fragmented forest, while in fragmented forest habitat, the annual home range for elephants is estimated to be around 600 km^2^.

The results also show that home range and movement rate for the elephants are influenced by the degree of habitat fragmentation. Once the key forest habitat for elephants is cleared, and the availability of the food plants and water sources altered, elephants are forced to expand and shift their ranges in the search for resources to meet their needs. Therefore, these findings could serve as a guideline where any habitat that is less than 500 km^2^ may not be suitable as a long-term territory for the Bornean elephant. Consequently any fragmented forest habitat (below 500 km^2^) should be connected to other continuous and large forest to secure a suitable area for the elephants to thrive.

The preferred habitat parameters for elephants were identified by this study to be non-fragmented dipterocarp forest, on flat land or with gentle slopes and below 300 meters elevation. Therefore, the lowland dipterocarp forests of Sabah are considered the most important habitat for the elephants, requiring high conservation protection.

As part of the recommendations to ensure the conservation of this species, two key recommendations are highlighted below: (i) all remaining lowland dipterocarp forests which support wild elephants should be retained under natural forest management and must not be converted to plantations; and (ii) forest disturbance needs to be minimized wherever wild elephants occur. In timber production forests, this can be achieved by limiting the extent and frequency of logging operations in any given management compartment.
